# Adolescent alcohol exposure alters threat avoidance in adulthood

**DOI:** 10.3389/fnbeh.2022.1098343

**Published:** 2023-01-25

**Authors:** Justine D. Landin, Lawrence Judson Chandler

**Affiliations:** Department of Neuroscience, Medical University of South Carolina, Charleston, SC, United States

**Keywords:** adolescence, alcohol, fear, platform-mediated avoidance, sex differences, foot shock, active avoidance, freezing

## Abstract

Adolescent binge-like alcohol exposure impairs cognitive function and decision making in adulthood and may be associated with dysfunction of threat avoidance, a critical mechanism of survival which relies upon executive function. The present study investigated the impact of binge-like alcohol exposure during adolescence on active avoidance in adulthood. Male and female rats were subjected to adolescent intermittent ethanol (AIE) exposure by vapor inhalation and then tested in adulthood using a platform-mediated avoidance task. After training to press a lever to receive a sucrose reward, the rats were conditioned to a tone that co-terminated with a foot-shock. A motivational conflict was introduced by the presence of an escape platform that isolated the rat from the shock, but also prevented access to the sucrose reward while the rat was on the platform. During the task training phase, both male and female rats exhibited progressive increases in active avoidance (platform escape) in response to the conditioned tone, whereas innate fear behavior (freezing) remained relatively constant over training days. A history of AIE exposure did not impact either active avoidance or freezing behavior during task acquisition. On the test day following platform acquisition training, female rats exhibited higher levels of both active avoidance and freezing compared to male rats, while AIE-exposed male but not female rats exhibited significantly greater levels of active avoidance compared to controls. In contrast, neither male nor female AIE-exposed rats exhibited alterations in freezing compared to controls. Following 5 days of extinction training, female rats continued to display higher levels of active avoidance and freezing during tone presentation compared to males. Male AIE-exposed rats also had higher levels of both active avoidance and freezing compared to the male control rats. Together, the results demonstrate that female rats exhibit elevated levels of active avoidance and freezing compared to males and further reveal a sex-specific impact of AIE on threat responding in adulthood.

## Introduction

Adolescence is a period of developmental change that includes increased risk-taking and sensation-seeking. It is therefore, not surprising that adolescence is also a period during which individuals typically begin experimenting with alcohol. A recent analysis of results from the Monitoring the Future National Survey reported that 3.4% of individuals aged 14 and 14% of individuals aged 18 reported having engaged in binge drinking behavior (defined as consumption of 5 or more drinks in a row) at least once within the past 2 weeks (Patrick and Terry-McElrath, 2019). In addition, a significant proportion of these individuals (1% of 14-year-old and 5.4% for 18-year-old individuals) also report having engaged in high-intensity binge drinking (defined as >10 drinks in a row) during this same time. Of particular concern are the potential long-term consequences of adolescent binge drinking on brain development. For example, increasing evidence from preclinical models demonstrates that a history of binge-like alcohol exposure during adolescence can lead to persistent changes in cognitive function in adulthood (Spear, 2018; Crews et al., 2019; Lees et al., 2020; Salmanzadeh et al., 2020; Sicher et al., 2022).

The ability to adaptively respond to threats in the environment is an important aspect of behavioral flexibility. While modulation of threat avoidance is essential for survival, an inability to appropriately evaluate and respond to threats is prevalent in many psychiatric disorders, including major depression and substance abuse (LeDoux and Daw, 2018; Mobbs, 2018). Preclinical models of Pavlovian auditory fear conditioning is a commonly used approach for investigating the role of learning and memory in threat responding that involves repeated pairing of an aversive stimulus (typically a foot shock) with a tone. After the formation of a learned association of the tone and the aversive shock, subsequent presentation of the tone alone elicits a defensive response. The most common readout of defensive responding in auditory fear conditioning is innate freezing behavior observed upon presentation of the fear-conditioned cue. However, while classic fear conditioning paradigms that assess freezing have been instrumental in advancing our understanding of fear learning and memory, this approach has a number of limitations for understanding the regulation of more complex forms of avoidance behaviors. Behavioral threat responding can be roughly categorized as being either goal-directed or non-goal-directed. Goal-directed avoidance is typically an action-outcome form of responding that is decision-based, while non-goal-directed responses are typically innate and passive in nature and include freezing and startle behaviors (LeDoux and Daw, 2018; Ball and Gunaydin, 2022). A threat response that involves performing a learned action to avoid harm is an active form of avoidance. This is in contrast to passive avoidance behaviors that do not require a response to avoid harm. In fact, in classic fear conditioning procedures, the innate freezing response has no impact on whether or not the animal will receive a shock.

The platform-mediated avoidance task (PMA) was developed as a means to access decision-based avoidance under conflict (Bravo-Rivera et al., 2014). An important feature of the PMA task is the incorporation of reward-seeking into the procedure that introduces a motivational conflict between reward and harm avoidance. While the animal learns that it can avoid a foot-shock by moving onto an escape platform in response to presentation of conditioned warning signal, it does so at the cost of forfeiting the opportunity to obtain a reward since it can physically no longer access the reward lever and reward magazine. The PMA task thus serves as a model of risky decision-making in which the animal must develop a response strategy that weighs the cost-benefits of obtaining a reward vs. harm avoidance. Since adolescent alcohol exposure has been previously shown to impact cognitive function and decision-making in adulthood (Spear, 2018; Crews et al., 2019), the present study utilized the PMA task to examine the effects of adolescent binge alcohol exposure on subsequent threat responding in adult male and female rats.

## Materials and methods

### Animals

Long-Evans dams were obtained from Envigo (Indianapolis, IN) and shipped with 10 pups of both sexes that were postnatal day (PD) 22 upon arrival. At PD 24, pups were weaned and pair-housed with same sex littermates in standard polycarbonate cages. Rats were maintained on a 12 h/12 h reverse light/dark cycle with *ad libitum* access to tap water and food (Teklad 2918, Envigo, Indianapolis, IN, USA) and water. All animal procedures were conducted in accordance with the National Institute of Health Guidelines under Institutional Animal Care and Use Committee approved protocols at the Medical University of South Carolina.

### AIE exposure

**Figure 1:**
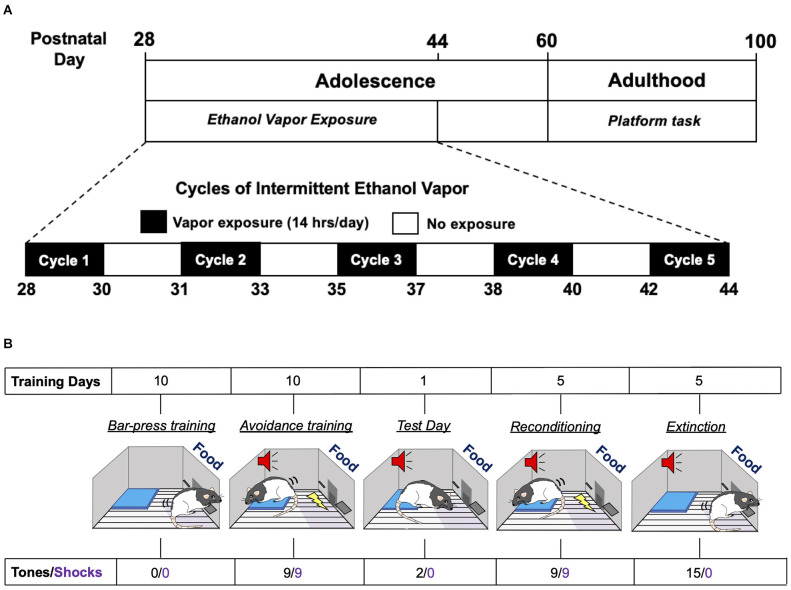
Experimental time-line of adolescent intermittent ethanol exposure and the platform-mediated avoidance task. **(A)** Schematic depiction of the daily sequence of adolescent intermittent ethanol (AIE) exposure by vapor inhalation followed by platform-media avoidance testing in adulthood. **(B)** Schematic depiction of the sequence for the different phases of the platform-mediated avoidance task for assessment of approach-avoidance behavior.

Adolescent male and female Long-Evans rats underwent five successive 2-day cycles of vapor ethanol exposure according to previously described methods (Gass et al., 2014). As depicted in Figure 1A, adolescent rats were subjected to five cycles of Air or ethanol vapor between PD 28–44, which is roughly equivalent to 10–18 years of age in humans (Spear, 2015). Each exposure cycle consisted of two consecutive days of intermittent exposure to ethanol vapor followed by two ethanol-free days. Each exposure day of a cycle consisted of 14 h in the vapor chambers followed by a 10 h ethanol-free period outside of the chamber. Each 2-day cycle was followed by 1–2 non-exposure day(s). Control rats were treated similarly to AIE-exposed rats but were only exposed to air. Behavioral intoxication scores were taken immediately upon the removal of the rats from the vapor chambers. Intoxication rating involved a subjective 5-point scale where 1 = no signs of intoxication; 2 = slight motor impairment; 3 = obvious motor impairment but able to walk; 4 = dragging abdomen, loss of righting reflex; and 5 = loss of righting and eyeblink reflexes (Nixon and Crews, 2002; Gass et al., 2014). Tail blood was collected immediately after intoxication scoring on the second day of each cycle for measurement of blood ethanol concentrations (BECs) using an Analox alcohol analyzer (Analox Instruments, Atlanta, GA).

### Platform-mediated avoidance (PMA) task

Threat responding was assessed using a platform-mediated avoidance (PMA) task as previously described (Bravo-Rivera et al., 2014). As depicted in Figure 1B, 1 week following AIE or Air exposure, rats were food restricted to 85%–90% of free-feeding body weight. On PD 60, rats began operant training to lever press for a reward using a variable interval schedule of reinforcement that averaged 30 s (VI30). During all phases of training and testing, sucrose pellets were available on the VI30 schedule. Rats were trained in standard operant chambers (29.53 × 24.84 × 18.67 cm; Med Associates, Burlington, VT, USA) located in sound attenuating cubicles (Med Associates). Reward lever training was conducted over a period of 7–10 days. Once a criterion of 10 lever presses/min was reached, platform-mediated avoidance training began. Three rats were excluded from the study because they did not meet the criteria.

During the platform-mediated avoidance training phase, rats were conditioned to a tone (30 s, 4 kHz, 75 dB) that co-terminated with a foot shock delivered through the metal floor grids (2 s, 0.4 mA). Tone-shock pairings were separated by a variable inter-trial interval that averaged 3 min. Rats received three tone-shock pairings per session, with a 5-min free-press interval in between sessions. Free-press intervals included time without shock or tone presentation while the reward was still available in order to reduce fear and suppression of lever pressing. Rats received three sessions per day, for a total of nine tone-shock pairings per day. During all phases of training and testing, including extinction, an immobilized acrylic square platform (14.0 × 14.0 × 0.33 cm) was located in the far corner of the floor opposite the active lever. During avoidance training, rats were able to avoid shock by moving onto the platform. Due to the platform’s location, rats must leave the safety of the platform in order to lever press to receive a sucrose reward pellet. Avoidance training was continued for a period of 10 days in order to attenuate freezing and lever press suppression when the tone was on. Active avoidance was defined as the rat having at least two paws on the platform and unable to reach the lever or food magazine. This was assessed at 1-s intervals across the entire period of tone presentation. On a subsequent retrieval test day (day 11), rats received two tones that did not co-terminate with shock. Rats were then re-conditioned to the tone-shock pairing and then underwent 5 days of extinction training with the platform in place, with each extinction session consisting of 15 tones per day that were not paired with shock. Testing and extinction days consisted of two or 15 sequential tone presentations, respectively, without any free-press intervals. At the beginning of lever press training, the average body weight of the rats was 267 g for the males and 204 g for the females. At the end of extinction training, the average body weight for the males was 324 g and 203 g for the females. There were no differences in body weights between the Air control and AIE groups for either sex.

### Data analysis

Data were recorded using digital cameras (Logitech, Newark, CA, USA), and freezing behavior was analyzed with AnyMaze software Version 7.00 (Stoelting Co., Wood Dale, IL, USA). The freezing function of AnyMaze automatically detects periods when the animal is freezing. The freezing score in AnyMaze is calculated using an “on” and “off” threshold. Using a training set of videos, the threshold parameters within AnyMaze were iteratively adjusted until automated freezing detection matched the freezing that was observed by visual inspection of the videos. The minimum duration for a freezing episode in AnyMaze was set at 750 ms. For active avoidance behavior, platform location detection in AnyMaze was confirmed by manually reviewing the video recordings to assess whether a rat was positioned “on” or “off” the platform during each second of the 30-s tones. A rat was assigned a score of 0% if it was “off” of the platform and a score of 100% if it was positioned “on” the platform. Data across time were averaged into 3-s bins and across each session per trial day. Unless otherwise stated, data were analyzed using a 2-way ANOVA with Sidak’s *post-hoc* comparisons. The average percent time rats spent on the platform during the 30-s tone was analyzed using a mixed-effects ANOVA. Analysis of the percentage of rats that remained on the platform during the shock interval (SI) at the end of the tone was analyzed using a chi-squared analysis. For these studies, in order to identify whether a rat was considered to have remained on or off the platform during the SI, rats that had received an average score higher than 50% were considered on the platform during the session. The percentage of rats that remained on the platform during the SI was compared across groups.

## Results

The present study utilized a well-characterized model of AIE exposure by vapor inhalation that is designed to simulate repeated episodes of binge intoxication. The procedure involved subjecting adolescent rats to intermittent cycles of ethanol vapor between PD28–44. Behavioral intoxication and blood ethanol concentration (BEC) were assessed at the end of each exposure cycle. The average intoxication score using the 1–5 point rating scale was 1.78 ± 0.10 for male rats and 2.16 ± 0.14 for female rats, which corresponds to a modest level of intoxication. The corresponding BEC values were 250 ± 12.0 mg/dl for the male rats and 246 ± 24.6 for the female rats. Statistical analysis (unpaired *t*-test) revealed that intoxication scores in the female rats were significantly higher than in the males (*p* = 0.0309), whereas there were no significant differences in the BEC values between the male and female rats.

At PD60, rats began training on the PMA task. During the 10 days of task acquisition training, active avoidance (platform escape) was assessed by calculating the average percent time the rats spent on the platform during a 30-s tone that predicted delivery of a shock during the final 2 s of the tone. For analysis of task acquisition by male rats (Supplementary Figure S1A), a mixed-effects ANOVA of active avoidance behavior (expressed as time spent on the platform) revealed a main effect of time (acquisition days) [*F*_(9,174)_ = 9.639, *p* < 0.001]. While there was a non-significant trend of treatment (Air vs. AIE) [*F*_(1,22)_ = 3.442, *p* = 0.077], there was a significant time × treatment interaction [*F*_(9,174)_ = 2.126, *p* = 0.029]. Freezing behavior was also assessed in the same animals during task acquisition by measuring the percent of time the rats froze during tone presentation (Supplementary Figure S1C). The ANOVA revealed there was no main effect of time [*F*_(9,174)_ = 0.940, *p* = 0.491] or treatment [*F*_(1,22)_ = 0.532, *p* = 0.473], nor was there a significant time × treatment interaction [*F*_(9,174)_ = 1.766, *p* = 0.077]. Analysis of total lever presses during each session across task acquisition (Supplementary Figure S1E) revealed a main effect of time [*F*_(9,174)_ = 8.568, *p* < 0.001]. There was no significant effect of treatment [*F*_(1,22)_ = 3.523, *p* = 0.073] or a time × treatment interaction [*F*_(9,174)_ = 1.187, *p* = 0.306].

For the acquisition of active avoidance behavior in the female rats (Supplementary Figure S1B), a mixed-effects ANOVA revealed a significant main effect of time [*F*_(9,201)_ = 5.842, *p* < 0.001], indicating that similar to male rats, Air and AIE-exposed female rats also increased their time spent on the platform during the tone at similar rates during task acquisition. There was no main effect of treatment [*F*_(1,25)_ = 2.978, *p* = 0.096] or of a time × treatment interaction [*F*_(9,201)_ = 1.727, *p* = 0.084]. Analysis of freezing across acquisition days by the female rats (Supplementary Figure S1D) revealed a significant main effect of time [*F*_(9,201)_ = 2.342, *p* = 0.015], but no significant effect of treatment [*F*_(1,25)_ = 0.5705, *p* = 0.457] or of a time × treatment interaction [*F*_(9,201)_ = 0.8990, *p* = 0.5271]. Analysis of total lever presses by the female rats during task acquisition (Supplementary Figure S1F) revealed a significant effect of time [*F*_(9,198)_ = 3.192, *p* = 0.001] and a time × treatment interaction [*F*_(9,198)_ = 2.008, *p* = 0.040], but no main effect of treatment [*F*_(1,25)_ = 0.036, *p* = 0.850].

Taken together, the above analyses revealed that both male and female rats exhibited progressive increases in the acquisition of active avoidance across training days as they learn to avoid the shock by retreating to the platform. In contrast, innate freezing behavior was relatively low from the outset and did not change across acquisition days. The results also indicate that a history of AIE did not impact either active avoidance or freezing behaviors during the task acquisition phase.

### Active avoidance and freezing during the test phase of the task

**Figure 2:**
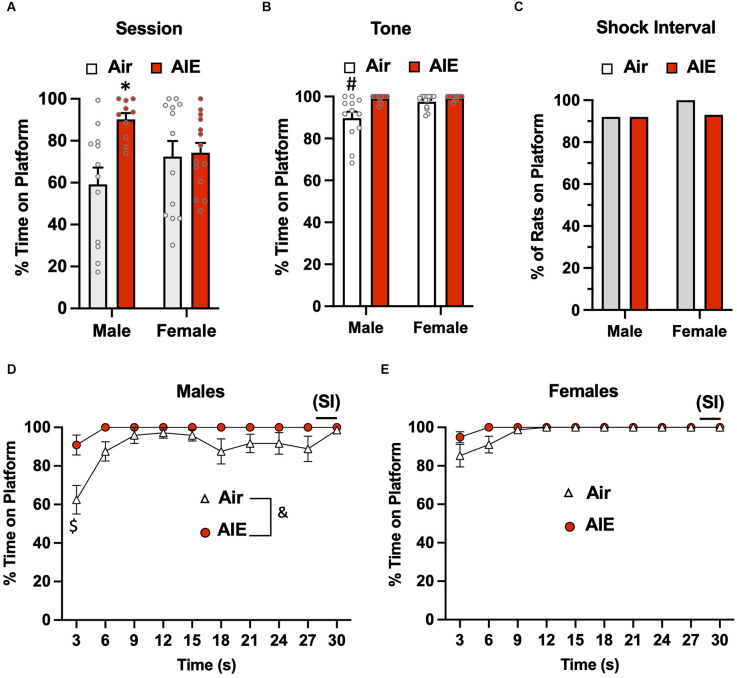
Active avoidance behavior assessed on the test day of the platform-mediated avoidance task. **(A)** When examined over the entire test session, AIE-exposed male rats spent significantly more time on the platform compared to the male Air control animals. **(B)** When the analysis of time on the platform was restricted to the periods of the 30-s tone, both male and female rats were located on the platform to a much greater extent compared to the tone-off periods. In addition, the significant difference in time on the platform between the male Air and male AIE-exposed rats was still observed. **(C)** Chi-square analysis revealed there was no significant difference in the percentage of rats that remained located on the platform during the 2-s shock interval (SI) at the end of the tone. Analysis of the time-course in 3 s bins of when the male **(D)** and female **(E)** rats were positioned on the platform during the tone revealed a significant main effect of AIE in the male rats. This was primarily driven by the significant difference observed at the initial 3 s time point. Data represent the means ± sem (except for the contingency date in panel **C**; *n* = 12–13/group). ^*^indicates significant difference from male Air rats, *p* = 0.013; ^#^indicates significant difference from all other groups, all *p* values < 0.01; ^$^indicates significant difference of Air and AIE at the 3 s time point, *p* = 0.000; ^&^indicates significant main effect of AIE, *p* = 0.009.

Following the 10-day task acquisition phase, rats underwent a single test session in which they were exposed to two tones that did not co-terminate with a shock. Analysis of the total percent time the rats were on the platform during this 5-min test session (Figure 2A) revealed there was a main effect of treatment (Air vs. AIE) [*F*_(1,45)_ = 6.669, *p* = 0.013], no main effect of sex [*F*_(1,45)_ = 0.042, *p* = 837], but a significant interaction between sex and treatment [*F*_(1,45)_ = 5.236, *p* = 0.027]. A Sidak’s multiple comparison *post-hoc* test revealed a significant difference between Air and AIE-exposed male but not female rats (*p* = 0.012). When the analysis was restricted to the time the rats were on the platform during the 30-s tone (Figure 2B), the ANOVA indicated there was a main effect of both treatments [*F*_(1,45)_ = 12.38, *p* = 0.001] and sex [*F*_(1,45)_ = 6.413, *p* = 0.014], and a significant treatment × sex interaction [*F*_(1,45)_ = 5.225, *p* = 0.027]. Sidak’s *post-hoc* test revealed that the time spent on the platform during the tone by the Air male rats was significantly different from all other groups (all *p* values < 0.01). A chi-squared analysis was used to assess the percentage of rats considered to be on the platform >50% of the time during the 2-s shock intervals (SI) averaged across each session (Figure 2C). This revealed that the percentage of rats on the platform during the SI was not different across the groups (*p* = 0.724).

We next examined the time-course of when the rats were on the platform during the tone (averaged across both tones). For the male rats (Figure 2D), a mixed-effect ANOVA revealed there was a main effect of time [*F*_(9,189)_ = 7.656, *p* < 0.001] and treatment (Air vs. AIE) [*F*_(1,21)_ = 8.272, *p* < 0.009], and a time × treatment interaction [*F*_(9,189)_ = 2.730, *p* = 0.005]. A Sidak’s multiple comparisons *post-hoc* test revealed that male AIE-exposed rats spent significantly less time on the platform compared to Air control rats during the first 3 s of the tone (*p* = 0.000). For the female rats (Figure 2E), the analysis revealed a significant main effect of time [*F*_(9,220)_ = 6.161, *p* = 0.000], but no main effect of treatment [*F*_(1,220)_ = 0.4865, *p* = 0.486] or of a time × treatment interaction [*F*_(9,220)_ = 0.4865, *p* = 0.882].

**Figure 3:**
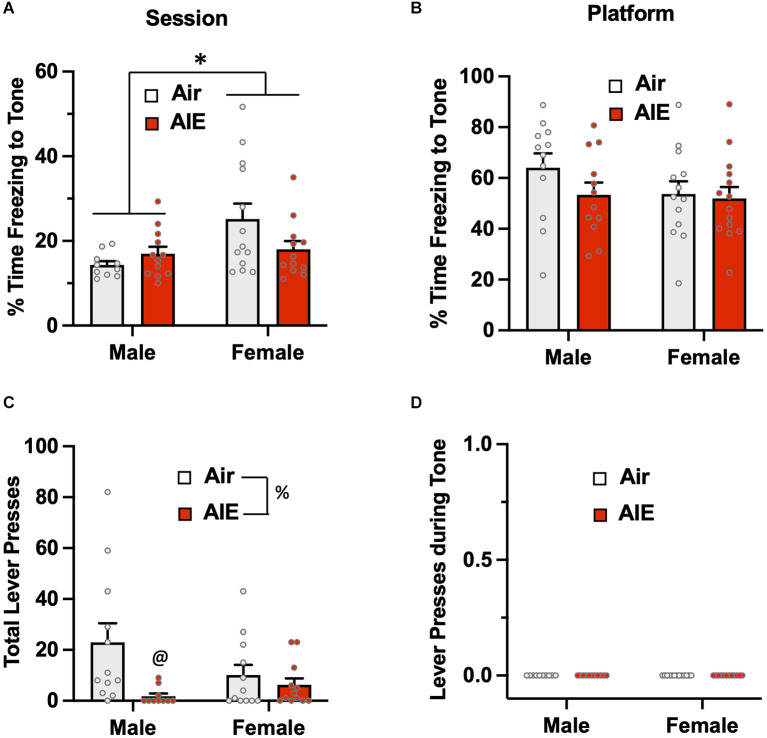
Freezing behavior and lever pressing assessed on the test day of the platform-mediated avoidance task. **(A)** When freezing to the tone on the test day was examined irrespective of location, female rats exhibited higher levels of freezing compared to the male rats. AIE exposure did not significantly alter freezing in either sex. **(B)** When analysis of freezing to the tone was restricted to when the rats were positioned on the platform, the sex difference in freezing was no longer observed. **(C)** Examination of lever responding during the test session revealed total lever pressing by AIE-exposed male rats was significantly reduced. **(D)** No lever pressing during the tone was observed. Data represent the means ± sem (*n* = 12–13/group). ^*^indicates significant main effect of sex, *p* = 0.02; ^@^indicates significant difference from the male Air rats, *p* = 0.010; ^%^indicates significant main effect of AIE, *p* = 0.010.

Passive avoidance behavior during the 5-min test session was assessed by measuring the percentage of time rats froze during the presentation of the two conditioned tones (Figure 3A). The ANOVA revealed there was a main effect of sex [*F*_(1,43)_ = 5.748, *p* = 0.020] in which female rats exhibited higher levels of freezing than males. While there was no significant main effect of treatment (Air vs. AIE) [*F*_(1,43)_ = 0.834, *p* = 0.366], there was a non-significant trend towards a sex × treatment interaction [*F*_(1,43)_ = 3.927, *p* = 0.053]. When analysis of freezing to the tone was restricted to when the rats were on the platform (Figure 3B), the ANOVA indicated there was no main effect of sex [*F*_(1,47)_ = 1.530, *p* = 0.222] or treatment [*F*_(1,47)_ = 1.377, *p* = 0.246], and no sex × treatment interaction [*F*_(1,47)_ = 0.083, *p* = 0.374]. Analysis of lever pressing during the 5 min test session (Figure 3C), revealed a main effect of treatment [*F*_(1,42)_ = 7.094, *p* = 0.010]. A follow-up multiple comparisons *post-hoc* test indicated that male AIE-exposed rats pressed the lever significantly less compared to the male Air control rats. While there was no main effect of sex on total lever presses [*F*_(1,42)_ = 0.800, *p* = 0.375], there was a non-significant trend of a sex × treatment interaction [*F*_(1,42)_ = 3.404, *p* = 0.072]. For lever pressing during the tone, there were no differences across sex or AIE exposure as no rats lever pressed during this period (Figure 3D).

Taken together, the above analysis of the test phase of the task indicates that female rats exhibit higher levels of both active and passive avoidance behavior compared to male rats. In addition, it further revealed that a history of AIE exposure is associated with sex-specific alteration in active but not passive avoidance.

### Active avoidance and freezing during the extinction phase of the task

**Figure 4:**
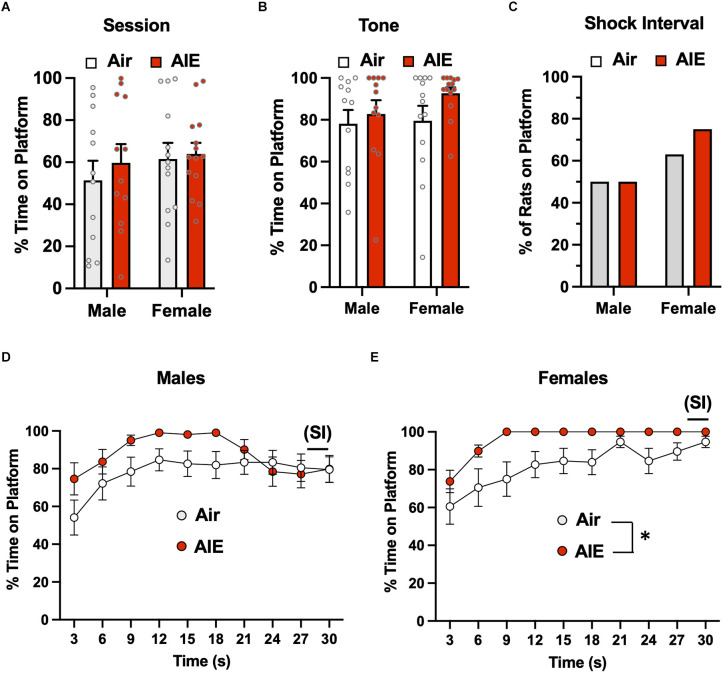
Active avoidance behavior assessed on the first day of extinction of the platform-mediated avoidance task. When the percentage of time the rats were positioned on the platform was examined over the entire session **(A)** or only during the tone presentations **(B)**, there were no significant difference across sex or AIE. **(C)** Chi-square analysis indicated there were no differences in the percentage of rats considered to have remained on the platform during the 2-s shock interval (SI). Analysis of the time-course in 3 s bins of when the male **(D)** and female **(E)** rats were on the platform during the tone presentations revealed a significant main effect of time and a significant main effect of AIE in the female rats. Data represent the means ± sem (except for the contingency date in panel **C**; *n* = 12–13/group). ^*^indicates a significant main effect of AIE, *p* = 0.046.

The third phase of the PMA task involved the examination of the extinction of avoidance behavior over five consecutive days, with each extinction day consisting of 15 tone presentations but no shock co-termination. For active avoidance on the first day of extinction (Figure 4A), ANOVA analysis of the percent time rats spent on the platform during the entire session revealed there was no main effect of sex [*F*_(1,47)_ = 0.8664, *p* = 0.356] or treatment [*F*_(1,47)_ = 0.4696, *p* = 0.496], nor was there a significant sex × treatment interaction [*F*_(1,47)_ = 0.1437, *p* = 0.706]. When the analysis was restricted to the time on the platform during the 30-s tone (Figure 4B), the ANOVA again revealed no main effect of treatment [*F*_(1,47)_ = 0.5062, *p* = 0.480] or sex [*F*_(1,47)_ = 0.9208, *p* = 0.342], nor was there a treatment × sex interaction [*F*_(1,47)_ = 0.5062, *p* = 0.480]. Chi-squared analysis of the percentages of rats on the platform during the 2-s SI averaged across each session (Figure 4C) also revealed no group differences (*p* = 0.507). For analysis of the time-course of when the male rats were on the platform during the 30-s tone (Figure 4D), there was a main effect of time [*F*_(9,186)_ = 8.461, *p* = 0.000] and a significant time × treatment interaction [*F*_(9,186)_ = 2.821, *p* = 0.003], but no main effect of treatment [*F*_(1,22)_ = 0.3997, *p* = 0.533]. With the female rats (Figure 4E), there was a main effect of time [*F*_(9,195)_ = 14.55, *p* = 0.000] and of treatment [*F*_(1,25)_ = 4.384, *p* = 0.046], but no time × treatment interaction [*F*_(9,195)_ = 0.7436, *p* = 0.668].

**Figure 5:**
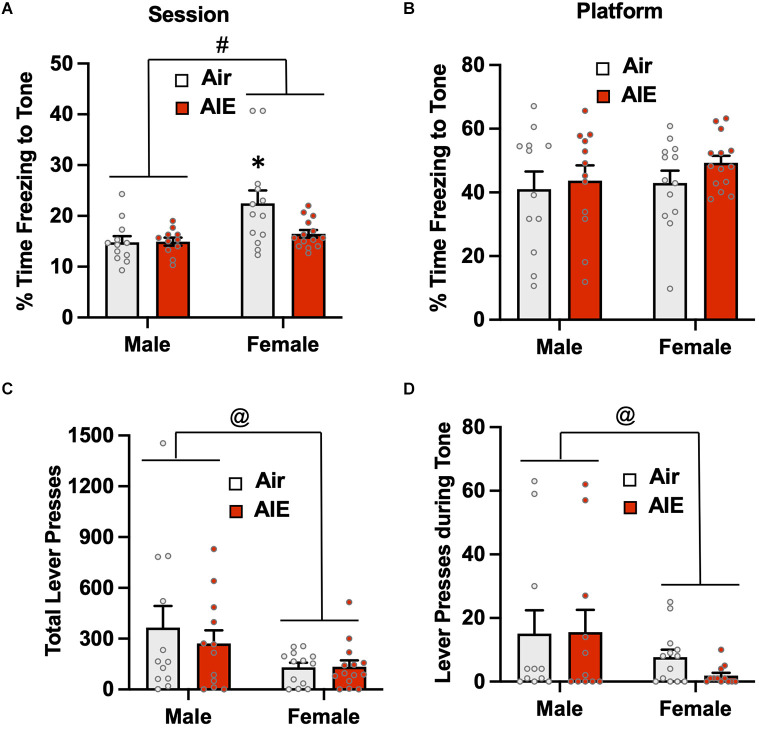
Freezing behavior and lever pressing assessed on the first day of extinction of the platform-mediated avoidance task. **(A)** When freezing during tone presentation on the first day of extinction was examined irrespective of the location, female rats exhibited higher levels of freezing compared to the males. A prior history of AIE exposure significantly reduced freezing to the tone in the female rats. **(B)** When analysis of freezing during tone presentation was restricted to when the rats were located on the platform, the sex difference and effect of AIE exposure on freezing was no longer observed. **(C)** Examination of total lever responding during the test session revealed lever pressing by female rats was significantly reduced compared to male rats. **(D)** The sex-specific reduction in lever pressing was also observed during presentation of the tone. Data represent the means ± sem (*n* = 12–13/group). ^#^indicates significant difference between males and females, *p* = 0.005; ^*^indicates significant difference from all other groups, all *p* values < 0.05; ^@^indicates significant differences between males vs. females, all *p* values < 0.05.

Analysis of passive avoidance behavior on the first day of extinction (Figure 5A) indicated there was a significant main effect of sex [*F*_(1,46)_ = 8.715, *p* = 0.005]. Follow-up *post-hoc* analysis revealed freezing to the tone by the female Air rats was significantly greater compared to all other groups (all *p* values < 0.05). While there was a trend towards a main effect of treatment [*F*_(1,46)_ = 3.850, *p* = 0.055] and towards an interaction of sex × treatment [*F*_(1,46)_ = 3.586, *p* = 0.064], neither reach the level of statistical significance. When the analysis was restricted to freezing while on the platform (Figure 5B), there was no main effect of sex [*F*_(1,47)_ = 0.826, *p* = 0.368] or treatment [*F*_(1,47)_ = 1.149, *p* = 0.289], and no treatment by sex interaction [*F*_(1,47)_ = 0.1956, *p* = 0.660]. Analysis of lever pressing during the Day 1 extinction session (Figure 5C) revealed a main effect of sex [*F*_(1,47)_ = 6.189, *p* = 0.016], but no effect of treatment [*F*_(1,47)_ = 0.3659, *p* = 0.548] or of a sex × treatment interaction [*F*_(1,47)_ = 0.4237, *p* = 0.518]. When this analysis was restricted to lever presses only during the tone (Figure 5D), there continued to be a main effect of sex [*F*_(1,43)_ = 4.520, *p* = 0.039], but no treatment [*F*_(1,43)_ = 0.2895, *p* = 0.593] or sex × treatment interaction [*F*_(1,43)_ = 0.3968, *p* = 0.532].

Overall, the above analysis of avoidance behaviors during the first day of extinction indicates that while a history of AIE exposure did not significantly impact passive avoidance on the first day of extinction, female rats tend to display higher levels of passive avoidance than their male counterparts and exhibit reductions in lever pressing to obtain a sucrose reward.

**Figure 6:**
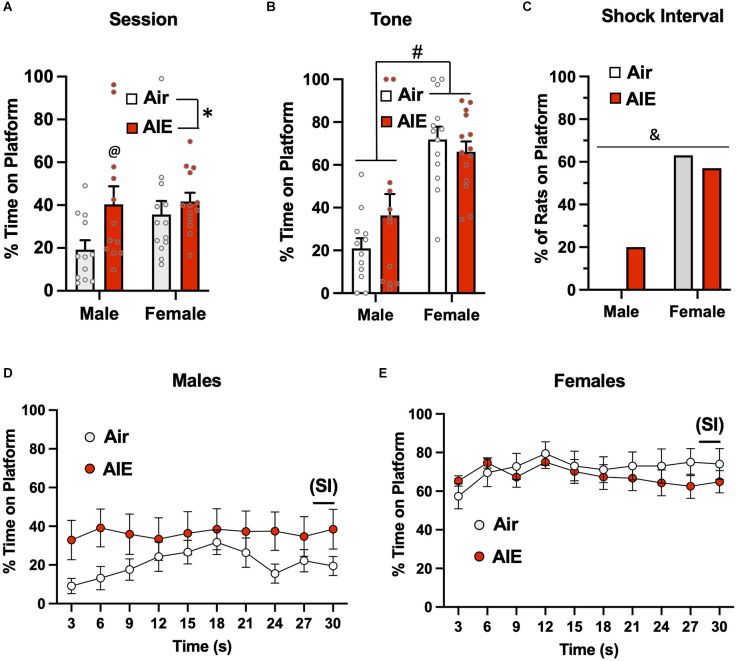
Active avoidance behavior assessed on the last day of extinction of the platform-mediated avoidance task. **(A)** When the percentage of time the rats were located on the platform was examined over the entire extinction session, male AIE-exposed rats exhibited significant increases in time on the platform compared to the male Air rats. **(B)** When analysis of time on the platform was restricted to the period of the tones, the female rats continued to spend significantly more time on the platform. **(C)** Chi-square analysis also revealed there was a significant difference in the percentage of rats considered to have remained on the platform during the 2-s shock interval (SI). Analysis of the time-course in 3 s bins of when the male **(D)** and female **(E)** rats were located on the platform during the tone presentations revealed a significant main effect of time in the female rats. Data represent the means ± sem (except for the contingency date in panel **C**; *n* = 12–13/group). ^*^indicates significant difference between Air and AIE, *p* = 0.028; ^@^indicates significant difference between the male Air and male AIE rats, *p* = 0.018; ^#^indicates significant difference between male and female rats, *p* = 0.000; ^&^indicates significant group difference, *p* = 0.000.

Analysis of the time spent on the platform during the entire session of the 5th day of extinction (Figure 6A) revealed a significant main effect of treatment [*F*_(1,46)_ = 5.137, *p* = 0.028], but no main effect of sex [*F*_(1,46)_ = 2.175, *p* = 0.147] or of a treatment × sex interaction [*F*_(1,46)_ = 1.550, *p* = 0.219]. Sidak’s *post-hoc* test indicated that the percent time spent on the platform by the male Air rats was significantly lower compared to the male AIE (*p* = 0.018) and female AIE rats (*p* = 0.011). When the analysis was restricted to the 30-s tone period (Figure 6B), the ANOVA again indicated a main effect of sex [*F*_(1,47)_ = 37.17, *p* = 0.000], but no main effect of treatment [*F*_(1,47)_ = 0.5348, *p* = 0.468] nor was there a treatment × sex interaction [*F*_(1,47)_ = 2.551, *p* = 0.116]. Sidak’s multiple comparisons *post-hoc* test revealed that both groups of male rats were significantly different from both groups of female rats (all *p* values < 0.01). Whether the rats were considered to have remained on the platform during the 2-s SI were averaged across each session for extinction day 5 (Figure 6C). A Chi-squared analysis revealed a significant difference in the percentage of rats on the platform during the shock interval (*p* = 0.000). Specifically, the percentage of rats from each group considered to be on the platform during the shock interval ranged from 0% for male Air rats, 20% for male AIE-exposed rats, 63% for female Air rats, and 57% for female AIE-exposed rats. Examination of the time-course of when male rats were on the platform during the 30-s tone (Figure 6D) revealed there was a non-significant trend of a main effect of time [*F*_(9,197)_ = 1.858, *p* = 0.060], and no significant effect of treatment [*F*_(1,22)_ = 1.974, *p* = 0.174] or of a time × treatment interaction [*F*_(9,197)_ = 1.602, *p* = 0.116]. With female rats, analysis of the time-course of when they were on the platform during the tone (Figure 6E) revealed a main effect of time [*F*_(9,117)_ = 2.032, *p* = 0.041], but no main effect of treatment [*F*_(1,13)_ = 0.7117, *p* = 0.414] or of a time × treatment interaction [*F*_(9,99)_ = 0.7732, *p* = 0.641].

The next set of analyses examined extinction-dependent changes in percent time on the platform in the Air control male and female rats as a function of Test day (baseline), Extinction Day 1, and Extinction Day 5 (Supplemental Figure S2). Mixed effect one-way ANOVAs with Tukey’s *post-hoc* tests revealed significant reductions in the percent time on the platform for both males and females when compared across the 3 days [Males: *F*_(1.979,21.77)_ = 10.19, *p* = 0.0008; Ext Day 5 significantly different from Ext Day 1 (*p* = 0.0130) and Test Day (*p* = 0.0049); Females: *F*_(1.526,18.31)_ = 8.929, *p* = 0.0035; Ext Day 5 significantly different from Test Day (*p* = 0.0066)] and when assessed specifically during tone presentation [Males: *F*_(1.313,14.44)_ = 71.93, *p* < 0.001; Ext Day 5 significantly different from both Test Day (*p* < 0.001) and Ext Day 1 (*p* < 0.001).

**Figure 7:**
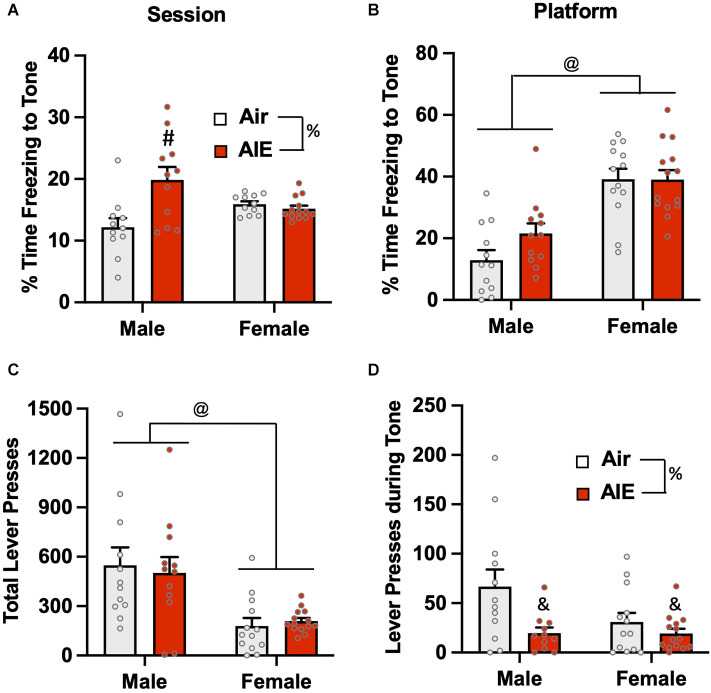
Freezing behavior and lever pressing on the last day of extinction of the platform-mediated avoidance task. **(A)** When freezing during tone presentation on the last day of extinction was examined irrespective of platform location, male AIE-exposed rats froze significantly more compared to male Air rats. **(B)** When analysis of freezing to the tone was restricted to the period when the rats were positioned on the platform, female rats froze significantly more than male rats. During the period, the significant increase in freezing in the male AIE-exposed rats compared to the male Air rats was no longer observed. **(C)** Examination of total lever responding during the test session revealed lever pressing by female rats was significantly reduced compared to male rats. **(D)** AIE-exposed male and female rats also exhibited reductions in lever pressing during presentation of the tone. Data represent the means ± sem (*n* = 12–13/group). ^%^indicates a significant main effect of AIE, all *p* values < 0.01. ^@^indicates a significant difference between male and female rats, *p* = 0.000; ^&^indicates significant difference of AIE group compared the their respective Air group, all *p* values < 0.05; ^#^indicates significant difference between mail Air and AIE rats, *p* = 0.002.

Analysis of the percent of time the rats froze to the tone on the last day of extinction (Figure 7A) revealed there was no main effect of sex [*F*_(1,42)_ = 0.1358, *p* = 0.713], but there was a main effect of treatment [*F*_(1,42)_ = 7.37, *p* = 0.009] and a treatment × sex interaction [*F*_(1,42)_ = 10.80, *p* = 0.002]. A Sidak’s multiple comparisons *post-hoc* test indicated that this effect was likely driven by increased freezing of male AIE-exposed rats compared to the male Air rats (*p* < 0.001). When the analysis was restricted to freezing during the tone while the rats were on the platform (Figure 7B), there was a main effect in the of sex [*F*_(1,47)_ = 44.91, *p* = 0.000], but no effect of treatment [*F*_(1,47)_ = 1.717, *p* = 0.196] or of a sex × treatment interaction [*F*_(1,47)_ = 1.829, *p* = 0.182]. Sidak’s multiple comparisons test revealed both groups of male rats froze significantly less than both groups of female rats (all *p* values < 0.01). Analysis of lever pressing during the Day 5 extinction session (Figure 7C) revealed that female rats lever pressed significantly less than male rats [*F*_(1,47)_ = 20.28, *p* < 0.001], but there was no effect of treatment [*F*_(1,47)_ = 0.01, *p* = 0.910] nor was there a sex × treatment interaction [*F*_(1,47)_ = 0.2721, *p* = 0.604]. A Sidak’s multiple comparisons *post-hoc* test revealed that lever pressing in both groups of male rats was significantly greater than lever pressing in both groups of female rats (all *p* values < 0.05). When the analysis was restricted to lever pressing during the tone (Figure 7D), there was a significant main effect of treatment [*F*_(1,46)_ = 8.014, *p* = 0.006], but no main effect of sex [*F*_(1,46)_ = 3.029, *p* = 0.088] nor was there a treatment × sex interaction [*F*_(1,46)_ = 2.936, *p* = 0.093]. *Post-hoc* analysis revealed that the AIE-exposed male (*p* = 0.017) and female (*p* = 0.009) rats exhibited significantly lower levels of lever pressing during the tone compared to their respective Air control groups.

The final set of analyses examined extinction-dependent changes in percent time freezing in the Air control male and female rats as a function of Test day (baseline), Extinction Day 1, and Extinction Day 5 (Supplementary Figure S3). Mixed effect one-way ANOVAs with Tukey’s *post-hoc* tests revealed no significant changes in freezing in either male or female rats when compared across days [Males: *F*_(1.409,13.39)_ = 1.392, *p* = 0.2726; Females: *F*_(1.394,23.69)_ = 2.868, *p* = 0.0922]. When assessed specifically during tone presentation, male but not female rats exhibited significant time-dependent reductions in freezing [Males: *F*_(1.767,19.44)_ = 28.38, *p* < 0.0001; Ext Day 5 significantly different from both Test Day (*p* = 0.0001) and Ext Day 1 (*p* < 0.0035), Test Day significantly different from Ext Day 1 (*p* = 0.0055)] [Females: *F*_(1.862,22.35)_ = 3.753, *p* = 0.4908].

Together, the results from analysis of the last day of extinction revealed significant differences in avoidance behavior as a function of both sex and a history of AIE. Both active and passive avoidance in female rats was extinguished to a much less degree than in the male rats, which was also reflected by a reduction in lever pressing for the reward. Conversely, AIE exposure reduced the extinction of both active and passive avoidance only in male rats.

## Discussion

Preclinical studies investigating the long-term effects of adolescent alcohol exposure indicate that adult rats that had been subjected to repeated episodes of binge-like alcohol intoxication during adolescence display signs of increased anxiety-like behaviors and deficits in decision-making (Nasrallah et al., 2009; Clark et al., 2012; Gass et al., 2014; Schindler et al., 2014; Pandey et al., 2015; Crews et al., 2016; Barker et al., 2017; Chandler et al., 2022). There is also evidence that certain types of experiences during critical periods of adolescence can alter brain development and circuit maturation and lead to permanent changes in adult behavior, including the expression of threat responding (Dow-Edwards et al., 2019; Gerhard et al., 2021). The present study examined the impact of adolescent binge-like alcohol exposure in male and female rats on behavioral responding in a reward-avoidance conflict task. This task required animals to balance the competing motivation to retrieve a reward against the motivation to avoid receiving a shock. Our results revealed that female rats exhibit elevated levels of both active avoidance and innate freezing behavior compared to male rats, and further revealed a sex-specific effect of AIE on threat responding in adulthood (summarized in Table 1).

**Table 1:** Summary of effect of sex and AIE exposure on active avoidance and freezing behaviors across acquisition, test day, and extinction.

Sex/Condition	Behavior	Acquisition	Test Day	Extinction day 1	Extinction day 5
Males:	Avoidance	-	↑	-	↑
Effect of AIE	Freezing	-	-	-	↑
Females:	Avoidance	-	-	-	-
Effect of AIE	Freezing	-	-	↓	-
Females compared	Avoidance	-	-	-	↑
to Males	Freezing	-	↑	↑	↑

*Significant increases or decreases as a function of treatment (AIE compared to Air) or sex (females compared to males) are indicated by upward or downward arrows, respectively (*p* < 0.05). Active avoidance represents changes time on the platform during the entire session or during the tone. Freezing represents changes in freezing during tone presentation across the entire session or while on the platform*.

The active avoidance task we employed involved an initial training period during which the rats learned to avoid a foot-shock by moving onto an escape platform in response to the presentation of a warning signal (tone). During training, both male and female rats rapidly acquired active avoidance as evidenced by a progressive increase in time spent on the platform during the presentation of the shock predicting tone. There were no differences between males and females or Air and AIE-exposed rats in the percentage of time spent on the platform, the percentage of time spent freezing, or the total number of reward lever presses. This lack of effect of AIE on these behavioral measures during task training is in agreement with previous studies indicating that AIE exposure minimally impacts basic learning processes (including responding to sucrose reward) that do not tax higher-order cognitive resources (Coleman et al., 2011; Semenova, 2012; Acheson et al., 2013; Risher et al., 2013; Gass et al., 2014; Towner and Spear, 2021). While studies using the PMA task have previously observed a reduction in freezing during the acquisition of platform avoidance (Bravo-Rivera et al., 2014), we did not observe changes across training in the percentage of time rats froze, nor did we see a change in total reward lever pressing across training. Since rats are unable to access the reward lever while positioned on the platform, it is interesting that there was not a reduction in reward lever pressing across training in spite of the progressive increase over training days in the time spent on the platform. A likely explanation for this is that the rats compensated for a reduction in the time of lever access by increasing reward-seeking during the inter-trial intervals, which may reflect the optimization of a reward-avoidance behavioral strategy.

Following the completion of active avoidance training, a test session was conducted to examine retrieval of the avoidance memory. Analysis of time spent on the platform during the entire test session or during the tone period only revealed that AIE-exposed male rats spent significantly more time on the platform compared to male Air control rats. In contrast, the percentage of rats on the platform during the shock interval indicated that all groups of rats equally remained on the platform during the period of time when the shock would have been delivered. The fact that AIE-exposed male rats spent the majority of their time on the platform during the retrieval test session is consistent with a significant reduction in reward-seeking in these rats compared to the male Air control rats. The time-course of platform escape during the conditioned tone also revealed treatment and sex differences in recall of learned active avoidance behavior. This difference was primarily observed during the initial period of the tone when the male Air rats displayed a delay in moving onto the platform. This was in contrast to the male AIE-exposed animals that either rapidly relocated to the platform upon tone presentation or were already positioned on the platform prior to initiation of the tone. As expected based upon the fact that the rats were on the platform nearly the entire time of the tone presentation, reward lever pressing during this period was virtually non-existent.

It is of interest that the results of previous studies regarding the effects of adolescent alcohol exposure on anxiety-like behaviors in adult rats appear to depend upon the strain of the rat being studied. For example, adult male Sprague-Dawley rats that had been subjected to AIE exhibited increased time in the closed arms of the elevated plus maze (EPM; Pandey et al., 2015; Kyzar et al., 2019), whereas adult male Wistar and Long-Evans rats that had undergone AIE exposure displayed increased time in the open arms of the EPM (Gilpin et al., 2012; Gass et al., 2014). A potential explanation for these seemingly opposing observations is that while AIE exposure may have enhanced anxiety across rat strains, there are strain-specific differences in their threat-coping mechanisms. The increase in open arm time on the EPM by AIE-exposed Long-Evans rats may reflect a bias towards disinhibition and increased escape responding. Such an effect would be consistent with the AIE-induced enhancement of active avoidance in the current study that was also carried out in Long-Evans rats.

Extinction of conditioned fear is a form of behavioral flexibility commonly investigated in animal models (Bouton et al., 2021). When a conditioned stimulus (e.g., auditory tone) is presented repeatedly in the absence of the unconditioned stimulus (e.g., foot-shock), extinction learning is observed as a gradual reduction of the conditioned response (Myers and Davis, 2007). Extinction is not simply the forgetting of a previously learned association, but instead involves the formation of a new extinction memory that competes with the original fear memory for the control of behavior (Quirk and Mueller, 2008; Gass and Chandler, 2013; Bouton et al., 2021). The relative strengths of the fear and extinction memories can be manipulated to influence which memory controls behavioral responding. For example, increasing the number of extinction trials increases the relative strength of the extinction memory compared to the strength of the original fear memory. In the case of the PMA task, there is also a motivational conflict between the desire to avoid a shock and the motivation to obtain a reward, and this motivational component can impact extinction learning. It should be noted that in order for the rats to undergo avoidance extinction in the PMA task, they must learn that the shock is no longer being delivered, which can only occur if the animal is not on the platform during the shock interval. Thus, the additional component of motivated reward-seeking in the PMA task may facilitate the rate of extinction learning by enhancing the incidence of platform off-time during the shock interval (i.e., more opportunities to extinguish the CS-US association). The fact that the percentage of rats on the platform during the shock interval rapidly declined on the first day of extinction (compared to the preceding test day) indicates that rats were able to quickly assess that a shock was no longer being delivered at the end of the conditioned tone.

Our results also reveal a striking difference in the extinction of active avoidance between male and female rats in the PMA task. On the first day of extinction training, both male and female Air control rats spent a similar percentage of time on the platform during the conditioned tone (78.1% for males and 79.5% for females). However, by the last day of extinction, male rats exhibited a large reduction in the percent time spent on the platform during the tone, while the percentage of time the female rats were on the platform did not significantly change (21.0% for males and 71.9% for females). These sex differences were also reflected in the percentage of rats that remained on the platform during the SI. The percentage of male Air control rats on the platform during the SI dropped from 50% on the first day of extinction to 0% on the last day of extinction. In contrast, the percentage of female Air control rats on the platform during the shock interval did not change during extinction training (63% on both the first and last day of extinction). Taken together, these observations indicate that female rats were more resistant to the extinction of active avoidance compared to male rats. While the percentage of time male and female Air control rats spent freezing during each daily session did not change across training, the percent freezing by males was significantly lower when they were on the platform. In contrast, there was no change in the freezing across extinction by females when assessed during either the session or while located on the platform. It should be noted that an interpretation of sex-dependent differences in the extinction of freezing behavior specifically during tone presentation may be confounded by the fact that male rats spent comparatively little time on the platform by the end of extinction training. Our results also revealed sex-specific effects of AIE exposure on extinction learning. While there were no differences in active avoidance between Air and AIE-exposed rats on the first day of extinction training, by the last day of training the AIE-exposed male (but not female) rats spent significantly more time on the platform compared to the Air male rats. A similar sex-specific effect was observed with the extinction of freezing behavior. Together, our observations suggest that AIE exposure promoted extinction-resistant avoidance behavior that was specific to males. However, the interpretation of a selective effect of AIE on extinction learning in males may also by confounded by the fact that female rats, regardless of treatment, were substantially more resistant to extinction.

As preclinical studies of fear-related behaviors have traditionally been carried out in male rats, there is a substantial gap in our knowledge of sex differences in threat responding. While the limited number of studies involving traditional fear-conditioning and extinction paradigms have yielded some conflicting conservations (Velasco et al., 2019), they tend to suggest that female rats display a reduction in the acquisition of contextual fear conditioning compared to males (Maren et al., 1994; Pryce et al., 1999; Wiltgen et al., 2001; Kosten et al., 2005; Gresack et al., 2009; Ribeiro et al., 2010). In agreement with this, we recently reported that female Long-Evans rats exhibited lower levels of freezing during fear conditioning, more rapid extinction of freezing behavior, and lower levels of freezing during fear recovery compared to male rats (Chandler et al., 2022). With active avoidance paradigms, some studies suggest that females rats may adopt more active coping strategies compared to male rats and that female rats exhibit an increase in risk-aversive behavior, which may promote increases in active avoidance compared to male rats (Beatty and Beatty, 1970; Steenbergen et al., 1990; Lalanza et al., 2015; Orsini et al., 2016, 2021; Shanazz et al., 2022). Although speculative, these increases may contribute to enhanced vulnerability to anxiety-related disorders observed in women (Li and Graham, 2017; Day and Stevenson, 2020). The results of the present study utilizing the PMA task are consistent with the idea that female rats exhibit increased active avoidance of a threat compared to male rats. Our results also suggest that AIE may have sex-specific effects on threat-coping strategies in males such that the AIE-exposed male adult rats resemble females in adopting higher levels of active avoidance behavior. However, a caveat to this interpretation is that there may have been a ceiling effect in female rats that prevented any further enhancement of active avoidance responding.

Heightened threat appraisal is thought to be a major driver of maladaptive behavior that includes extinction-resistant avoidance. Enhanced fear and anxiety, such as has been observed following AIE, have been associated with overestimation of the danger posed by a threat (Ball and Gunaydin, 2022). Therefore, the interpretation of extinction-resistant avoidance exhibited by the male rats could also have been confounded by an apparent increase in threat avoidance that was observed on the test day prior to the initiation of extinction training. Thus, an increase in threat appraisal in association with a stronger avoidance memory might be expected to be more difficult to extinguish. However, the fact that both Air and AIE-exposed rats were equally on the platform during the SI may argue against an AIE-induced increase in threat appraisal. In addition to threat appraisal, another potential contributing factor in the apparent expression of extinction-resistant avoidance could be the development of habitual avoidance behavior (Pittig et al., 2020; Manning et al., 2021; Ball and Gunaydin, 2022). Habitual avoidance can emerge during extended periods of training if the avoidance response is repeatedly reinforced, as might occur across the 10-day training period of the PMA task. Using a modified version of the PMA task that involved “extinction-with-response prevention,” it was recently reported that rats that had undergone prolonged avoidance training displayed habit-like impairments in extinction avoidance (Martinez-Rivera et al., 2020). We and others have previously shown that AIE exposure facilitates the development of habitual responding for a natural reward in female but not male rats (Barker et al., 2017; Towner and Spear, 2021). It is tempting to speculate that the extinction-resistant avoidance that we observed in female rats compared to male rats may reflect the development of habitual avoidance across the 10-day training period. However, while it is difficult to disentangle the relative contributions of threat appraisal and habitual avoidance to extinction given the experimental design of the PMA task used in the current study, the fact that the percent time freezing to the tone on the last day of extinction was greater in the female rats compared to the male rats may argue for heightened threat appraisal instead of increased habitual behavior as a contributing factor to extinction-resistant avoidance of the female rats.

In summary, the present study demonstrated that female rats exhibit elevated levels of active avoidance and freezing compared to males, and a sex-specific impact of AIE on threat responding in adulthood. These observations add to a growing body of evidence obtained from both human and animal studies that adolescent alcohol exposure can result in long-term alterations in adult behavior.

## Data availability statement

The original contributions presented in the study are included in the article/Supplementary material, further inquiries can be directed to the corresponding author.

## Ethics statement

The animal study was reviewed and approved by Institutional Animal Care and Use Committee approved protocols at the Medical University of South Carolina.

## Author contributions

LC and JL jointly designed the experiments, performed the statistical analysis, interpreted the data, generated the figures, and wrote the manuscript. JL performed the experiments. All authors contributed to the article and approved the submitted version.

## Supplementary material

SUPPLEMENTARY FIGURE 1 Active avoidance and freezing behavior assessed across the acquisition period of the platform-mediated avoidance task. **(A,B)** Male and female rats exhibited progressive increases in active avoidance (percent time located on the platform) over the time-course of training days. In contrast, the percent time freezing during tone presentation **(C,D)** and lever pressing **(E,F)** remained relatively stable over the time-course of training. A history of AIE exposure had no effect on either active or passive avoidance, or on lever pressing across training days. Data represent the means ± sem (*n* = 12–13). ^*^indicate a significant main effect of time (acquisition days), all *p* values < 0.01.

SUPPLEMENTARY FIGURE 2 Time-course of reduction in active avoidance across extinction training in male and female Air rats. Male and female rats exhibited progressive reductions in percent time on the platform when assessed across baseline (Test day), Extinction Day 1, and Extinction Day 5. This reduction was observed in the percent time on the platform during the entire session **(A,B)** and during the tone presentation period only **(C,D)**. Data represent the means ± sem (*n* = 12–13). ^%^indicates significantly different from all other days, all *p* values < 0.05; ^*^indicates significantly different from Test day only, all *p* values < 0.05; ^#^indicates significantly different from all other days, all *p* values < 0.001.

SUPPLEMENTARY FIGURE 3 Differential changes in freezing across extinction training in male and female control rats. When assessed during the entire session, there were no significant changes in percent freezing during tone presentation when accessed across baseline (Test day), Extinction Day 1, and Extinction Day 5 in either male **(A)** or female **(B)** rats. When was assessed when the rats were located on the platform, freezing during tone presentation was significantly reduced in male **(C)** but not female **(D)** rats. Data represent the means ± sem (*n* = 12–13). ^*^indicates significantly different from all other days, all *p* values < 0.001.
