# Supplementary figures and images for: Adolescent alcohol exposure alters threat avoidance in adulthood
Source: Front Behav Neurosci. 2023 Jan 25;16:1098343. doi: 10.3389/fnbeh.2022.1098343 (PMC9905129; doi:10.3389/fnbeh.2022.1098343)

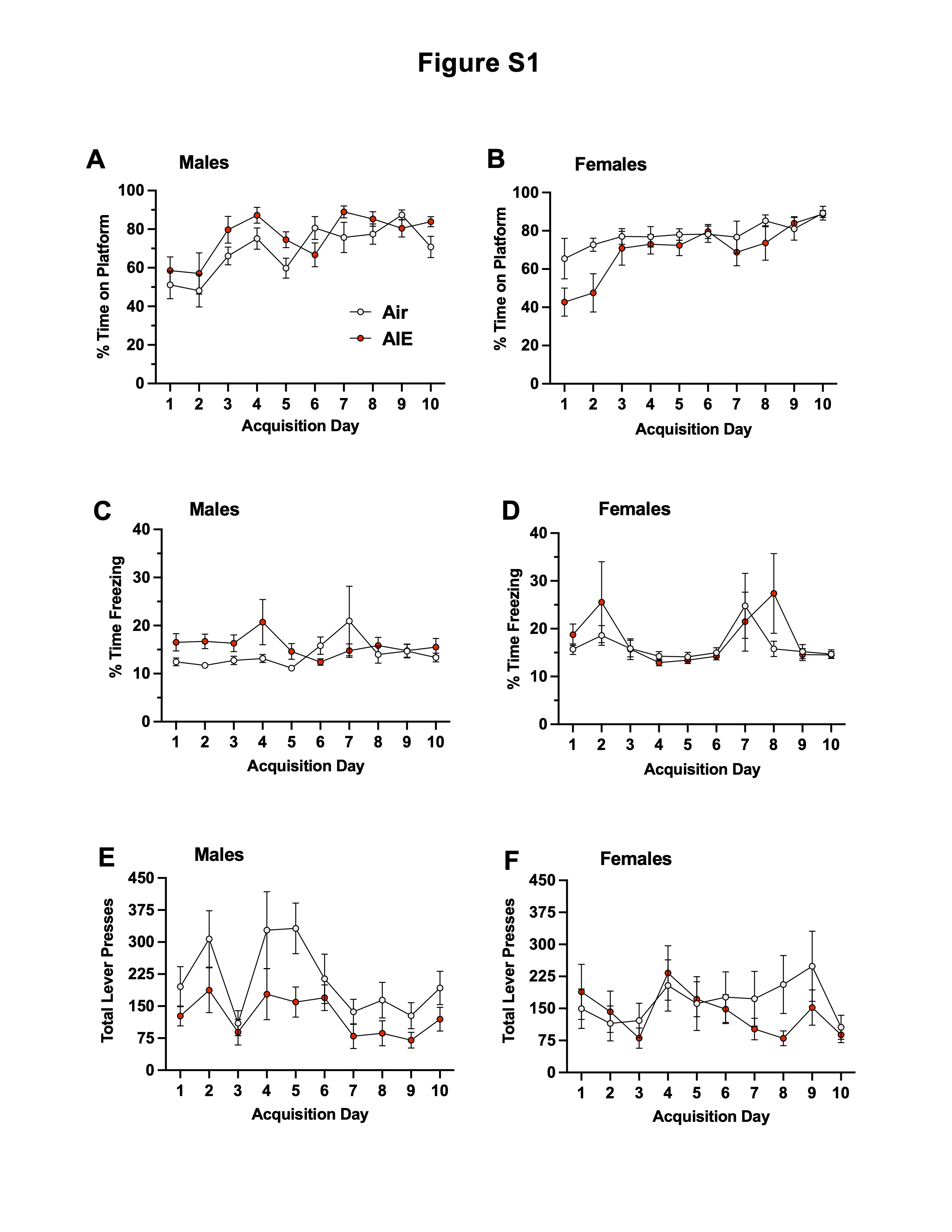

Supplement: SUPPLEMENTARY FIGURE 1 — Active avoidance and freezing behavior assessed across the acquisition period of the platform-mediated avoidance task. (A,B) Male and female rats exhibited progressive increases in active avoidance (percent time located on the platform) over the time-course of training days. In contrast, the percent time freezing during tone presentation (C,D) and lever pressing (E,F) remained relatively stable over the time-course of training. A history of AIE exposure had no effect on either active or passive avoidance, or on lever pressing across training days. Data represent the means ± sem (n = 12–13). *indicate a significant main effect of time (acquisition days), all p values < 0.01. [file Image_1.JPEG]

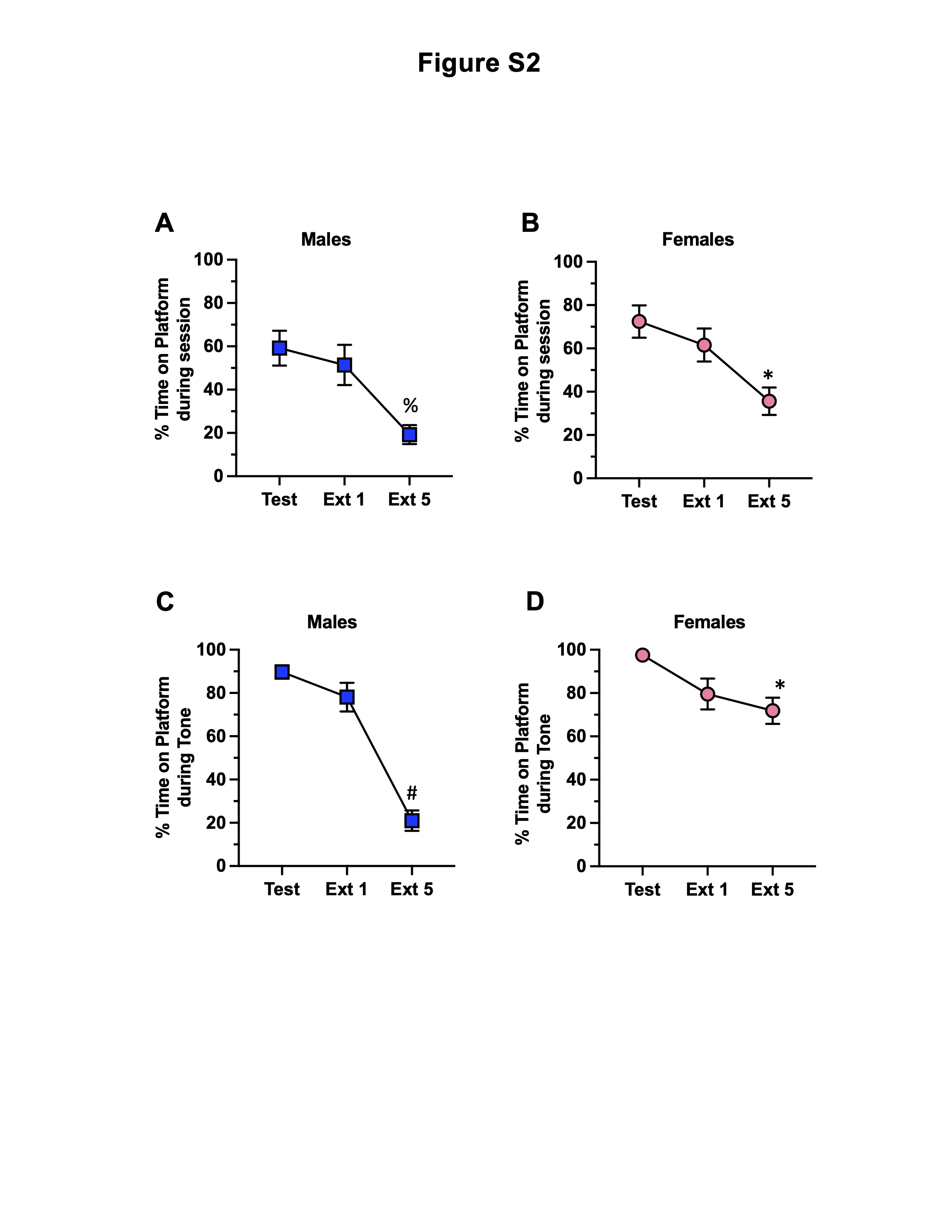

Supplement: SUPPLEMENTARY FIGURE 2 — Time-course of reduction in active avoidance across extinction training in male and female Air rats. Male and female rats exhibited progressive reductions in percent time on the platform when assessed across baseline (Test day), Extinction Day 1, and Extinction Day 5. This reduction was observed in the percent time on the platform during the entire session (A,B) and during the tone presentation period only (C,D). Data represent the means ± sem (n = 12–13). %indicates significantly different from all other days, all p values < 0.05; *indicates significantly different from Test day only, all p values < 0.05; #indicates significantly different from all other days, all p values < 0.001. [file Image_2.JPEG]

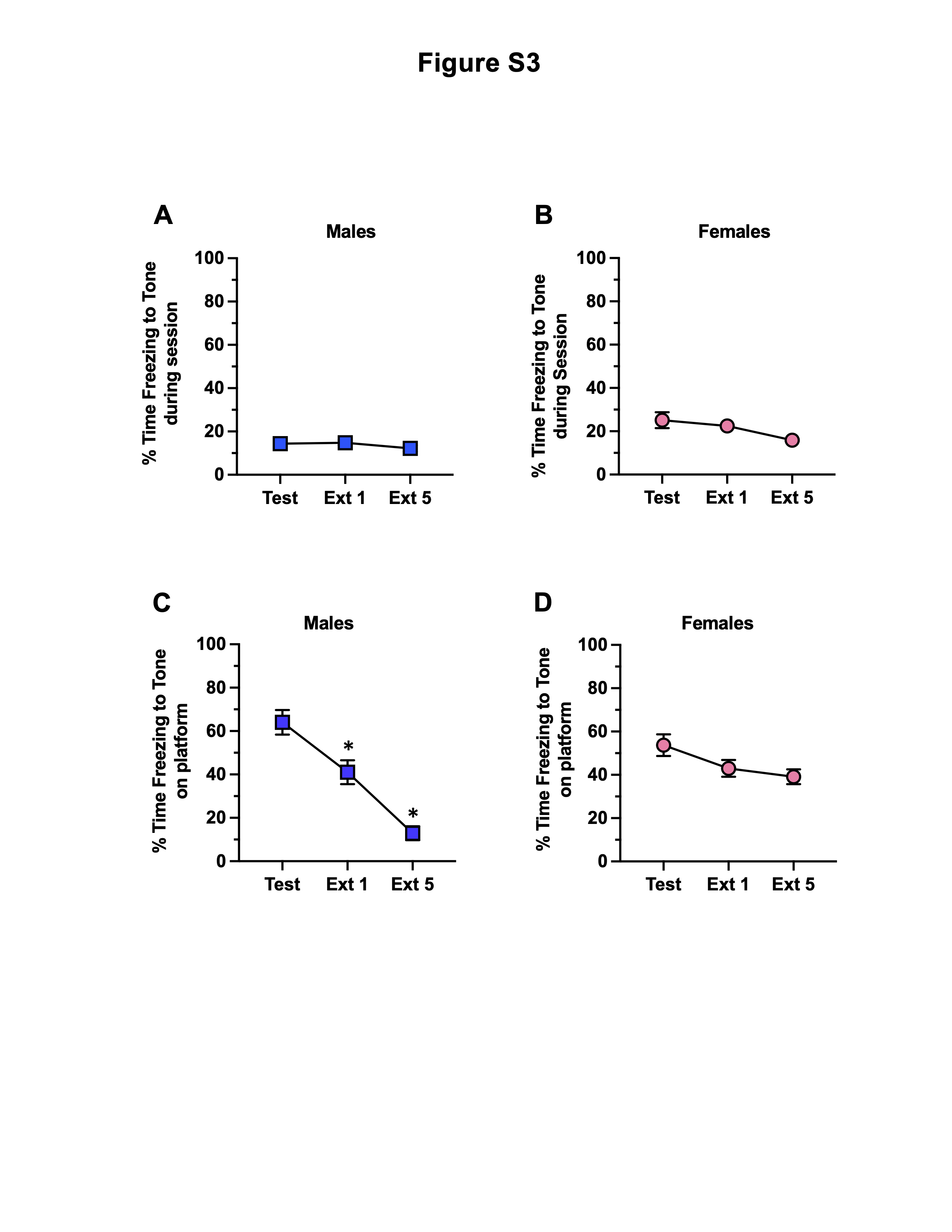

Supplement: SUPPLEMENTARY FIGURE 3 — Differential changes in freezing across extinction training in male and female control rats. When assessed during the entire session, there were no significant changes in percent freezing during tone presentation when accessed across baseline (Test day), Extinction Day 1, and Extinction Day 5 in either male (A) or female (B) rats. When was assessed when the rats were located on the platform, freezing during tone presentation was significantly reduced in male (C) but not female (D) rats. Data represent the means ± sem (n = 12–13). *indicates significantly different from all other days, all p values < 0.001. [file Image_3.JPEG]
